# CD4^+^FOXP3^+^ regulatory T cells are abundantly present in inflamed muscle of patients with juvenile dermatomyositis

**DOI:** 10.1186/1546-0096-9-S1-P63

**Published:** 2011-09-14

**Authors:** Yvonne Vercoulen, Elisabeth F Elst, Jenny Meerding, Maud Plantinga, Hemlata Varsani, Christa van Schieveen, Mette H Bakker, Mark Klein, Wim Spliet, R de Weger, Lucy Wedderburn, Annet van Royen- Kerkhof, Berent J Prakken

**Affiliations:** 1 Center for Molecular and Cellular Intervention, Pediatric Immunology, Wilhelmina Children’s Hospital, UMC Utrecht, The Netherlands; 2 University College London, Institute of Child Health, London, UK; 3 Department of Pathology, UMC Utrecht, The Netherlands

## Background

Juvenile dermatomyositis (JDM) is an autoimmune disease caused by inflammation of the microvasculature, resulting in skin and muscle damage. CD4^+^CD25^+^FOXP3^+^ regulatory T cells (Treg) are key regulators of immune homeostasis, and potential therapeutic targets in T cell-driven inflammatory disorders. However, a role for Treg in JDM pathogenesis has not yet been established.

## Aim

Here, we explored Treg presence and function in both peripheral blood mononuclear cells (PBMC) and muscle tissue of JDM patients.

## Methods

We analyzed expression of CD4, CD25, FOXP3, GITR and CTLA-4 in PBMC from JDM patients and age-matched controls by flow cytometry. Treg functionality was established in in vitro suppression assays. Infiltrated lymphocytes in muscle biopsies were analyzed for CD3, CD4, CD8 and FOXP3 expression by immunohistochemistry, qPCR (FOXP3) and flow cytometry (CD3 and CD4).

## Results

Overall, Treg number and phenotype of JDM patients was similar to age-matched controls. While JDM patients had increased numbers of Treg during active disease compared to remission, this increase was only found in JDM patients treated with high doses of corticosteroids. Treg of remitting JDM patients, but not of all active JDM patients were able to suppress effector T cell activation in vitro. Furthermore, in inflamed muscle tissue we detected high numbers of CD4^+^ T cells, of which a large proportion expressed FOXP3.

## Conclusion

Treg in peripheral blood of JDM patients had a similar phenotype and frequency as peripheral blood Treg from age-matched controls. Moreover, Treg were present in inflamed muscle tissue, and could therefore be targets for specific treatment of disease.

